# Linezolid Resistance in Staphylococci

**DOI:** 10.3390/ph3071988

**Published:** 2010-06-24

**Authors:** Stefania Stefani, Dafne Bongiorno, Gino Mongelli, Floriana Campanile

**Affiliations:** Department of Microbiology, University of Catania, Via Androne 81, 95124 Catania, Italy; E-Mails: d.bongiorno@unict.it (D.B.); ginomongelli@hotmail.it (G.M.); f.campanile@unict.it (F.C.)

**Keywords:** Staphylococci, Linezolid, mutations, *cfr*

## Abstract

Linezolid, the first oxazolidinone to be used clinically, is effective in the treatment of infections caused by various Gram-positive pathogens, including multidrug resistant enterococci and methicillin-resistant *Staphylococus aureus*. It has been used successfully for the treatment of patients with endocarditis and bacteraemia, osteomyelitis, joint infections and tuberculosis and it is often used for treatment of complicated infections when other therapies have failed.

Linezolid resistance in Gram-positive cocci has been encountered clinically as well as *in vitro*, but it is still a rare phenomenon. The resistance to this antibiotic has been, until now, entirely associated with distinct nucleotide substitutions in domain V of the 23S rRNA genes. The number of mutated rRNA genes depends on the dose and duration of linezolid exposure and has been shown to influence the level of linezolid resistance. Mutations in associated ribosomal proteins also affect linezolid activity. A new phenicol and clindamycin resistance phenotype has recently been found to be caused by an RNA methyltransferase designated *Cfr*. This gene confers resistance to lincosamides, oxazolidinones, streptogramin A, phenicols and pleuromutilins, decrease the susceptibility of *S. aureus* to tylosin, to josamycin and spiramycin and thus differs from *erm* rRNA methylase genes.

Research into new oxazolidinones with improved characteristics is ongoing. Data reported in patent applications demonstrated that some oxazolidinone derivatives, also with improved characteristics with respect to linezolid, are presently under study: at least three of them are in an advanced phase of development.

## 1. Introduction

**Figure 1:**
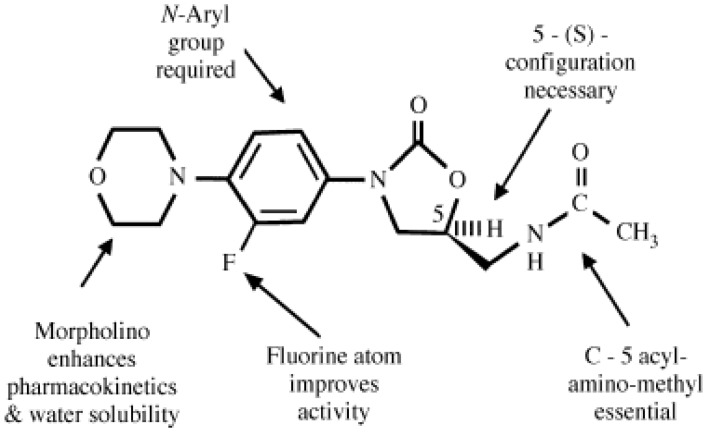
Structure-activity relationship leading to the development of linezolid [2].

Oxazolidinones represent a landmark in antimicrobial research being the first new class of antibiotics to enter clinical usage within the past 30 years. They were discovered by DuPont Pharmaceuticals in the late 1980s, but the early lead analogues (DuP 105 and DuP 721) proved unsuitable for pharmaceutical development and the program was dropped. Investigation was re-initiated by the then Upjohn Corporation in the early 1990s, leading to the delineation of a series of structure–activity relationships and to the synthesis of non-toxic analogues with good antibacterial activity. Although both eperezolid and linezolid showed excellent *in vitro* activity against Gram-positive bacteria, linezolid (PNU-100766, Figure 1) was chosen for further clinical development because of its superior bioavailability and improved serum levels, which allowed twice-daily dosing [1]. Consequently, only linezolid progressed to the subsequent phases of development [2]. 

## 2. Linezolid: Spectrum of *in Vitro* Activity

Linezolid has excellent *in vitro* activity against all of the major Gram-positive bacteria that are pathogenic in humans. Of these pathogens, 90% or more are inhibited by 4 mg of linezolid per L or less: the susceptibility breakpoint for staphylococci established by the U.S. Food and Drug Administration (FDA) [3]. For *Streptococcus pneumoniae* and other streptococci, a breakpoint of 2 mg/L or less for susceptible strains has been set. For enterococci, 2 mg/L or less indicates susceptibility, 4 mg/L indicates intermediate susceptibility, and 8 mg/L or greater indicates resistance (this resistance value is common to other species). The U.S. Clinical Laboratory Standards Institute (CLSI) and the European Agency EUCAST has established similar breakpoints [4,5].

Linezolid demonstrates *in vitro* activity against *Neisseria gonorrhoeae* and *Neisseria meningitidis*. It has only borderline activity against *Haemophilus influenzae* and is inactive against Enterobacteriaceae and *Pseudomonas* species [6,7]. Gram-negative bacilli are probably intrinsically resistant because they possess efflux pumps that are effective against linezolid [8]. Linezolid possesses activity against ''atypical organisms'', including *Legionella pneumophila*, *Mycoplasma pneumoniae*, and *Chlamydia pneumoniae*, and has good activity against many Gram-positive anaerobes. It is of interest that linezolid exhibits relatively good *in vitro* activity against many strains of *Mycobacterium tuberculosis* and is active against the *Mycobacterium avium* complex and several rapidly growing mycobacteria, including *Mycobacterium fortuitum*, *Mycobacterium chelonae*, and *Mycobacterium abscessus* [9,10]. Many clinical data on the activity of linezolid against mycobacteria have been published, some of them raising concerns about possible toxicity, especially hematologic and neuronal, when the drug was administered for long periods, but, this *in vitro* activity has stimulated research into oxazolidinone derivatives with even greater activity against these species [11]. Linezolid has also excellent *in vitro* activity against *Nocardia* species (including *Nocardia asteroides*, *Nocardia farcinica*, *Nocardia brasiliensa*) [12].

Linezolid (Zyvox®, Pfizer, in most countries) was approved by the FDA in 2000 for adults, and for pediatric use in 2005. It has been approved for the treatment of patients with community-acquired and nosocomial pneumonia, complicated skin and soft tissue infections and infections due to vancomycin-resistant *Enterococcus faecium*, or for the treatment of heteroresistant vancomycin-intermediate *Staphylococcus aureus* and penicillin-resistant pneumococci. Finally, it has been used successfully for the treatment of patients with endocarditis and bacteriemia, osteomyelitis, joint infections, and tuberculosis. It is often used for treatment of complicated infections when other therapies have failed [13,14,15]. 

## 3. The Mechanism of Action of Oxazolidinones

Oxazolidinones are inhibitors of ribosomal bacterial protein synthesis. Early biochemical studies suggested binding to the 30S ribosomal subunit or to the areas of the 50S subunit not previously implicated in substrate binding. More recently, by using an *in vivo* cross linking approach, structural information contradicted these previously *in vitro* result, showing that oxazolidinones bind with high affinity and great specificity to the catalytic site on the 50S subunit, at the ribosomal peptide-transferase centre, thus, affecting tRNA positioning [16,17,18].

Although the PTC is one of the most conserved regions of the ribosome, it is the target of several different antibiotics and by analyzing crystal structures of complexes of large ribosomal subunits from the *Deinococcus radiodurans* (used as a suitable model) with clinically useful antibiotics *i.e.* phenicols, lincosamides, pleuromutilins, streptogramins_A_ and oxazolidinones, different mechanisms were found. Chloramphenicol and linezolid clearly hamper A site tRNA binding (the site of entry of the aminoacyl tRNA); streptogramins_A_ and pleuromutilins hamper A and P site tRNAs accommodation, and clindamycin interferes with peptide bond formation [19]. Recent studies demonstrated that the binding of linezolid stabilizes the nucleobase U2585 (*E. coli* numbering) in a orientation that is distinctly different from when A and P-site tRNA ligands are bound, suggesting that linezolid induces a non-productive conformation of PTC [18]. Oxazolidinones also bind LepA, a universal bacterial elongation factor that back-translocates ribosomes from a post-translocation state to a pre-translocation state [20]. Although translation is recognized as the main target of oxazolidinones, there still remains some controversy as to the step at which inhibition takes place [18]. Recent data demonstrated that they can affect translational accuracy, promoting frameshifting and stop codon readthrough as the oxazolidinones do not inhibit peptide bond transfer [21].

Due to this mechanism of action, linezolid has a target that does not overlap with those of existing protein synthesis inhibitors, consequently, its activity is unaffected by the rRNA methylases that modify the 23S rRNA to block the binding of macrolides, clindamycin and group B streptogramins [22]; second, linezolid seems particularly effective in preventing the synthesis of staphylococcal and streptococcal virulence factors (e.g., coagulase, haemolysins and protein A), perhaps because of this mode of action [23]. 

Linezolid, like chloramphenicol, clindamycin, macrolides and tetracyclines, is essentially bacteriostatic [2], however, the drug exhibits *in vitro* killing (albeit slower than for most bactericidal agents) against streptococci, including *S. pneumoniae*, and *S. aureus* [1,6]. It is interesting to note that oxazolidinones bind only to the mitochondrial 70S ribosome and not to the cytoplasmic 80S ribosomes, explaining the myeolosuppression and toxic optic neuropathy observed in patients treated with prolonged use of linezolid [16,24,25,26].

## 4. Mechanisms of Resistance: in General

One of the critical advantages of linezolid over currently used antibiotics is its entirely synthetic nature. This means that it does not have a natural prototype, and, due to its characteristics, it was expected that there would be no natural pool of resistance genes which could facilitate the development of clinical resistance. It is well known that all other inhibitors of protein synthesis are derived from natural antibiotics of microbial origin whose producers serve as the natural reservoirs of resistance genes that can be transferred through horizontal gene transfer to clinical pathogens [15]. This advantage, until recently, was maintained – in fact, the only mechanism of resistance reported was due to mutations in the drug target site, primarily the rRNA of the large ribosomal subunit. This type of resistance, as described below, appears rarely, develops slowly because of the redundancy of rRNA genes in bacteria, and is not transferable between pathogenic species. This resistance was apparently generated *de novo* through spontaneous mutation rather than via genetic exchange.

The recent discovery of a mechanism of linezolid resistance based on acquisition of a natural and potentially transferable resistance gene that modifies a specific rRNA nucleotide located in the site of the drug action, is of particular concern and could completely change the picture of linezolid susceptibility in the future. This gene is apparently associated with mobile genetic elements which raises the possibility of its transmission both intra-species and to other pathogenic strains. 

Generally speaking it is difficult to induce resistance to linezolid. It is possible, however, to produce mutants. The reason is because the PTC is highly conserved, and altering the identity of PTC nucleotides in the immediate vicinity of the antibiotic is unfavorable; common mechanisms for acquiring resistance are based on altering the conformation and the flexibility of remote nucleotides: in fact, it was demonstrated that approximately half of the nucleotides mediating antibiotic resistance reside at distances >6 Å [19]. Resistance mediated by remote mutations can also include mutations in ribosomal proteins such as L4, and in the recently discovered L3 [27]. However, resistance can be acquired also by alterations of nucleotides that interact with the drug, with a mechanism that can be considered an alternative to the previous one. 

U2504 plays an important role in resistance to PTC antibiotics because it belongs to the binding pockets of phenicols, lincosamides, pleuromutilins and oxazolidinones. Mutations of U2504, due to this key position in the PTC center, are expected to cause serious problems to cells, consequently, altering neighbouring nucleotides (second and third layer nucleotides) that can remotely affect U2504, circumvent its essentiality. The role of this mutation was originally detected in *H. halobium* [28]. Furthermore, the involvement of the same nucleotides in resistance to several antibiotic families of different chemical nature occurs presumably because of the overlapping binding sites of these drugs. Because only a limited pool of nucleotides belonging to the PTC rear wall and the tunnel entrance is used for acquiring resistance, the probability of inducing resistance to more than a single antibiotic family by altering a given nucleotide is fairly high. This effect is further enhanced by the potential flexibility and the central location of U2504, which amplifies its possible involvement in resistance to various PTC antibiotics by indirect perturbation of its conformation and flexibility [19].

Further to these mechanisms, linezolid resistance together with resistance to clindamycin and tiamulin, were observed experimentally in *E. coli* strains, due to post transcriptional modifications: one specific post transcriptional modification in the PTC—*i.e.* conversion of U2504 to pseudouridin, notably increases cell resistance to several antibiotics targeting the large ribosomal subunit [29]. The lack of pseudourudin at position 2504 was found to significantly increase the susceptibility of bacteria to peptidyl transferase inhibitors.

In addition to all mechanisms described above, a recent paper, using a whole genome sequencing of three independent Lin^R^ *S. pneumoniae* (lab mutants), demonstrated clearly that other than the already described G2576T mutation, there was also the involvement of ABC proteins—the *spr*0333—corresponding to an rRNA methyltransferase modifying the G2445 residue [30]. Table 1 summarizes the main mechanisms currently found in Gram-positive cocci.

Three classes of oxazolidinone resistance mechanisms have been characterized: mutations in the domain V region of 23S rRNA genes; acquisition of the ribosomal methyltransferase gene *cfr*; and mutations in *rpl*D, and *rpl*C genes that encode 50S ribosomal proteins L4, and L3 respectively. 

## 5. Resistance in Domain V of the 23S rRNA and Related Proteins.

Over the past 10 years, an increasing number of isolates that are resistant to macrolide, lincosamide, streptogramin, ketolide, and oxazolidinone (MLSKO) antibiotics have been identified which contain mutations in domain V of the 23S rRNA genes, and/or the genes coding the ribosomal proteins L4 and L22 [31]. The majority of telithromycin (ketolide) and/or linezolid resistant bacteria carry mutations in one of these three genes. These mutational changes have been described in both Gram-positive and Gram-negative bacteria and alter the function of the 23S rRNA and/or proteins resulting in moderately decreased susceptibility to one or more of the MLSKO antibiotics [22]. Resistance has been associated with mutations in the central loop of the domain V region. Nearly all bacteria have multiple copies of the 23S rRNA gene, which was thought to make the development of resistance to these agents less likely [32]. The G2576T transversion (*Escherichia coli* 23S rRNA gene numbering), is responsible for the resistance to linezolid in microrganisms including *S. aureus*, CoNS, viridans group streptococci, *Enterococcus faecium,* and *E. faecalis* [13,33]. Tsiodras *et al.* reported in 2001 the first clinical isolate of linezolid-resistant *S. aureus*, which contained a G2576T mutation in the domain V region of the 23S rRNA gene [34]. In a follow-up study, this isolate was found to have five copies of this gene, each of which contained the G2576T mutation [35]. The number of rRNA genes mutated depends on the duration of linezolid exposure and its dose, and has been shown to influence the level of linezolid resistance [36].

**Table 1:** Mechanisms of linezolid resistance in Gram-positive cocci.

Genetic mechanisms	Site mutations or ribosomal protein mutations*	Microrganisms	References
Mutations in domain V	G2576T	*S. aureus*	[34]
		CoNS	[13]
		Viridans streptococci	[35]
		Enterococci	[37]
		*S. cohnii*	[38]
		*S. simulans*	Personal data—Stefani *et al.* ms. in preparation
		*S. hominis*	Personal data—Stefani *et al.* ms. in preparation
	G2505A	Enterococci	
		*S. aureus*	[39]
	G2512T	Enterococci	[39]
	G2513T	Enterococci	[39]
	C2610G	Enterococci	[39]
	G2447T	*S. aureus* (lab mutant)	[39]
	T2500A	*S. aureus*	[37]
			[36]
	C2192T	*S. aureus*	[40]
	G2447T	*S. aureus*	[41]
	A1743T	*S. pneumoniae*	[30]
	A2503G	*S. aureus*, *S. pneumoniae* (lab mutant)	[42]
			[30]
	T2504C	*S. aureus*	[42]
		*S. epidermidis*	[43]
	G2766T	*S. aureus*	[44]
	G2631T	*S. epidermidis*	[43]
	C2543T	*S. epidermidis*	[43]
	C2576T	*S. epidermidis*	[43]
Mutations in rplD (L4 r-protein) *	_65_WR_66_ and _68_KG_69_ deletions	*S. pneumoniae*	[45]
	A202C	*S. aureus* (lab mutant)	[46]
			[47]
	K68N, L108S, N158S substitutions	*S. epidermidis*, *S. pneumoniae*	[43]

	_71_GR_72_ and _71_GGR_72_ insertions		[45]
Mutations in rplC (L3 r-protein) *	G455A, G463C	*S. aureus*	[46]
	A505T, Δser145		
cfr Methyltransferase	A2503	*S. aureus*	[48]
		*S. epidermidis*	[49]
			[50]
			[51]
		*S. sciuri*	[52]

* E. coli numbering.

Pillai *et al*. described a series of increasingly linezolid-resistant MRSA isolates that contained increasing numbers of mutant (G2576T) copies of the 23S rRNA gene. In this study of laboratory-derived linezolid-resistant *S. aureus* isolates, it was demonstrated that MICs increased in proportion to the number of copies of mutations in the 23S rRNA genes [35]. Similar mutant-gene dosage effects have been seen in laboratory-derived oxazolidinone-resistant *S. aureus* mutants and in clinical isolates of linezolid-resistant enterococci [37,53]. 

Other mutations involving the domain V region of the 23S rRNA gene have been reported among laboratory derived from linezolid-resistant enterococci (G2505A, G2512T, G2513T, and C2610G), *S. aureus* (G2447T), *Escherichia coli*, *Mycobacterium smegmatis*, and *Halobacterium halobium* [28,54,55].

Meka *et al*. analyzed sequential clinical *S. aureus* isolates that became resistant to linezolid after several months of exposure to the drug. They detected for the first time the presence of a T2500A mutation in the domain V region of the 23S rRNA gene, along with the loss of a single copy of this gene in the more-resistant isolates [36,37]. Despite evidence of fitness costs associated with some 23S rRNA mutations [56,57,58], highly linezolid resistant 23S rRNA homozygous mutant strains of *S. aureus*, *S. epidermidis*, and *E. faecalis* have been recovered clinically. Even if, to date, G2576T and T2500A are the most common mutations found in clinical isolates, a variety of 23S rRNA mutations conferring resistance to linezolid have been identified, including C2192T [40], G2447T [41], A2503G [42], T2504C [42], G2505A [39], G2766T [44], and G2576T [34].

A less common mechanism of linezolid resistance involves mutations in ribosomal protein L4, which is encoded by the *rpl*D gene, and L3, encoded by the *rpl*C gene. These two mechanisms, previously identified in streptococci, were recently associated to linezolid resistance in staphylococci of clinical origin (see table 1) [27]. With regard to the possible involvement of the L22 ribosomal protein encoded by the *rpl*V gene, even if it is located far from the Lin binding site, it has already been described as responsible for resistance to quinupristin/dalfopristin in *S. pneumoniae* and *S. aureus* [59,60]. This target, together with many others ribosomal proteins, cannot be *a priori* excluded and needs further investigations, due to the presence of “unknown” resistance mechanisms reported in literature [55,61,62,63].

## 6. The *Cfr* Mechanism

A new phenicol and clindamycin resistance phenotype has recently been found to be caused by an RNA methyltransferase designated *Cfr*. A detailed analysis by drug footprinting studies and matrix-assisted laser desorption–ionization time of flight/tandem mass spectrometry, showed that *Cfr* adds an additional methyl group at position A2503 of 23S rRNA. Since A2503 is located in proximity to the overlapping ribosomal binding sites of phenicols and clindamycin, it was concluded that the *Cfr*-mediated methylation confers resistance to these two classes of antimicrobial agents by interfering with the positioning of the drugs [48]. This gene conferred resistance to phenicols, lincosamides, oxazolidinones, pleuromutilins and streptogramin_A,_ (known with the acronym of PhLOPSa_A_), but not to macrolides, and thus differs from *erm* rRNA methylase genes [22] in which the methylation occurs in position A2058 [64,65].

The *cfr* gene was first discovered in 2000 during a surveillance study for florfenicol resistance among staphylococci from animals. It was initially detected on the 16.5-kb multiresistance plasmid p*SCFS1* from a bovine strain of *Staphylococcus sciuri* [52] and has also been found in bovine strains of *Staphylococcus simulans* [66]. In addition to *cfr*, the p*SCFS1* plasmid of the bovine *S. sciuri* strain, carries the rRNA methylase gene *erm*B (33), the aminocyclitol phosphotransferase gene *spc*, and the ABC transporter gene *lsa*(B), which confer resistance to macrolide-lincosamide-streptogramin_B_ (MLS_B_) antibiotics, spectinomycin, and lincosamides, respectively. The *cfr* gene was recently detected on the 35.7-kb plasmid p*SCFS3*, from a porcine *Staphylococcus aureus* strain, together with the chloramphenicol/florfenicol exporter gene *fex*A [67]. Cloning of the *cfr* gene and expression in *Escherichia coli* revealed that *Cfr* conferred resistance not only to the original Gram-positive hosts but also to Gram-negative bacteria [68]. Comparison with other protein sequences deposited in databases showed that the *Cfr* protein is not related to other known resistance-conferring rRNA methyltransferases but rather to the Radical SAM (*S*-adenosylmethionine) superfamily [48], which includes a wide range of enzymes from a diverse set of bacteria involved in radical protein formation, isomerization, sulfur insertion, anaerobic oxidation, and unusual methylations [48].

As stated before, the *cfr* gene was detected in *Staphylococcus* spp. of animal origin in Europe [52]. The *cfr* gene was also recently found in *Staphyloccoccus* isolated from human strains [15,49]. During the 2007 LEADER program, two linezolid resistant *Staphylococcus* strains were found: *S. aureus* (004-737X) and *S. epidermidis* (426-3147L). The *cfr* gene was found in both isolates and its structure was characterized by Mendes *et al*. downstream of the *cfr* gene, the presence of Δ*tnp*B was noted in the *S. aureus* isolate, which was identical to the structure described for the *pSCFS3* plasmid found in an *S. aureus* isolate collected from the respiratory tract of a pig [49] (AM086211). The DNA sequence upstream of the *cfr* gene in the *S. aureus* isolate showed the presence of *ist*AS and *ist*BS genes, which were also identical to those of the p*SCFS3* plasmid, suggesting that these insertion sequences may be involved in the mobilization of the *cfr* gene [50]. However, the *tnp*A gene, which was located further upstream of the *cfr* gene on the p*SCFS3* plasmid, yielded a negative result, suggesting that the upstream region of *cfr* on this isolate, significantly differed from that of the p*SCFS3* plasmid [49]. In this *S. epidermidis* strain only the *cfr* gene was found [49]. 

The *cfr* was recently found in Germany in MRSA ST398 and ST9 lineages that have their main reservoir in swine, but can colonize and cause infections in humans [69]. Another report described the detection of *cfr* in an *S. aureus* strain recovered from a clinical human isolate (designated CM-05) from Colombia. The MRSA CM-05 isolate was characterized, and it was found that, unlike the animal isolates, the gene was located in the chromosome, but it is probable that it was a part of an integrated plasmid possibly capable of excision and mobilization [15]. Also in MRSA CM-05, the *cfr* gene was clustered in the chromosome with the *erm*(B) gene (which encodes another rRNA methylase and that confers resistance to macrolide, lincosamide, and streptogramin_B_ antibiotics), forming a transcriptional unit designated the *mlr* (for modification of the large ribosomal unit) operon, which is controlled by the *erm*B promoter. Despite the presence of putative regulatory short open reading frames, both genes are expressed costitutively. The combined action of the two methyltrasnferases encoded by the *mlr* operon result in modification of two specific residues in 23S rRNA, A2058 and A2053; this made the MRSA isolate resistant to all antibiotics whose target is the large ribosomal subunit [15,36]. In agreement with this finding, the plasmid from the CM05 isolate failed to make recipient *S. aureus* cells resistant to linezolid. The *erm*B/*cfr* cluster is flanked on one side by the transposase/cointegrase gene *ist*AS from the IS*21*-*558* mobile genetic element. PCR analysis showed the presence of the complete IS*21*-*558*. The IS*21*-*558* element was shown to be implicated in the mobility of the *cfr* gene in animal isolates and might contribute to its mobilization in the clinical strain [50]. Upstream from *erm*B, a 5' segment of the gene *rep*S is present. Its product, the RepS protein, is involved in initiation of plasmid replication. A close association of the *cfr* gene with a characteristic plasmid gene indicates that integration of a plasmid carrying the *cfr* gene in the chromosome of CM05 cells was the likely route of acquisition of linezolid resistance by the MRSA human isolate [15]. The identification of the *cfr* gene in this isolate recovered from a patient after a short exposure to linezolid indicates that the gene was also most likely acquired by this microrganism under a selective pressure that did not involve exposure to oxazolidinones [15]. An alternative explanation is that the strain was selected in an unidentified patient exposed to linezolid and was then passed on to the case patient [36].

**Figure 2:**
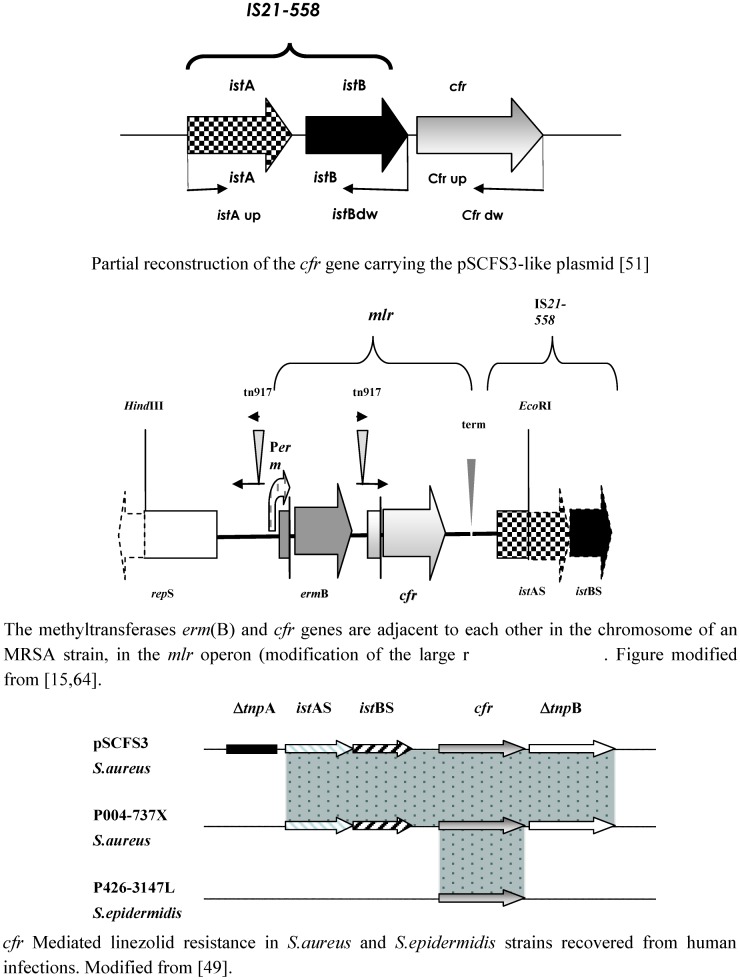
Genetic structures carrying the *cfr* gene.

After the detection of *cfr* in six staphylococcal isolates of animal origin, and the detection of this gene in sporadic clinical isolates of *S. aureus* and *S. epidermidis*, an outbreak of linezolid resistant *S. aureus* carrying the *cfr* gene was recently described in Spain [70] and the presence of *cfr* in bloodstream isolates of *S. epidermidis* was also recently documented in Italy. In these strains the *cfr* gene was located on a plasmid that is still being studied (personal communication, data not shown) [51]. Numerous other reports of linezolid resistant isolates have recently been demonstrated [71,72,73,74,75]. Figure 2 shows the molecular characteristics of mobile genetic elements carrying the cfr gene in clinical isolates [15,51,64]. 

## 7. Epidemiological Data on *S. aureus* and CoNS

Surveillance studies indicate that linezolid resistance is still extremely rare in both MRSA and CoNS clinical isolates. Recent epidemiological data show that linezolid resistance occurs in ≤1% of *S. aureus* isolates and ≤0.1% of CoNS in the US [62,76]. A study performed for monitoring emergence of linezolid resistance in isolates from 16 countries found only few CoNS strains were found, all carrying the G2576T mutation [63].

A frightening scenario can be foreseen if the appearance and spread of the *Cfr* methyltransferase parallels the situation observed for the Erm methyltransferase and combined resistance to MLS_B_. The *cfr* gene has been identified on structurally related multiresistance plasmids from animal staphylococci and can, in principle, be easily disseminated among staphylococci. However, surveillance studies in Germany have identified only six *cfr*-carrying staphylococcal strains over the past 17 years [77]. Linezolid resistance mediated by the presence of *Cfr* is still anecdotal, but clinicians should be aware of potential dissemination from animals to humans due to the ability for horizontal gene transfer to occurring between staphylococcal and enterococci isolates of both animals and humans [36].

## 8. Problems with *in vitro* Detection of Resistance

*In vitro* linezolid susceptibility can be determined by disk diffusion, broth microdilution, agar dilution, Etest, and most automated antimicrobial testing systems. The Etest method (AB Biodisk, Solna, Sweden) yields MICs that are typically one doubling dilution lower than broth microdilution results, probably because the manufacturer recommends reading and endpoint at 90% inhibition instead of complete inhibition [2,78]. 

Even if the disk diffusion method was effective in detecting the first reported isolates of *S. aureus* that were non-susceptible to linezolid [34], the results using these agar-based methods could be interpreted as either susceptible or non susceptible, depending on where the endpoints were read.

Tenover F. *et al*. [79], comparing the most commonly used susceptibility testing methods (manual and automated) challenged with linezolid-non susceptible staphylococci, found 25 major errors among the 297 results reported for staphylococci. With different levels of performance among the six methods compared in the paper (overall essential agreement was as follows: MicroScan < Phoenix < Vitek2 = Etest < Vitek), generally the problem was much greater in the non-detection of resistance rather than a possible overcalling of resistance. The authors concluded that testing of linezolid against staphylococci must be added to the growing list of challenges for antimicrobial susceptibility testing methods.

## 9. Oxazolidinones: the Future

The area of oxazolidinone research is very active because of the emergence of MRSA and MDR resistant Gram-positive cocci. There is a great medical need for finding newer oxazolidinones with improved potency, aqueous solubility and reduced toxicity. In these attempts, modifications of the A- B-C-rings of linezolid have been reported [80]. Among all these compounds, some of them derived from C-ring modifications were identified as possible clinical candidates.

Radezolid (RX-1741) is a novel oxazolidinone with broader spectrum of coverage and increased activity against Gram-positive organisms as compared to other oxazolidinones. Radezolid has recently successfully completed two Phase 2 clinical trials: one for community acquired pneumonia (CAP) and the second for uncomplicated skin and skin structure infections (uSSSI). Phase 2 data obtained to date indicate that this compound is well tolerated and effective in the treatment of uncomplicated skin infections and in the treatment of community-acquired pneumonia. Rib-X (New Haven, Connecticut, USA) is developing both the oral and intravenous formulations of radezolid. It has been shown to be microbiologically more active than linezolid against Gram-positive organisms, including having potent activity against linezolid resistant bacteria, and intracellular species [81,82].

Torezolid (TR-700), developed by Trius Therapeutic Inc. (San Diego, CA, USA), is a second-generation oral and IV generation antibacterial drug in this class with activity against drug-resistant, gram-positive bacterial pathogens, including those resistant to linezolid. TR-700 is the active moiety of a novel oxazolidinone phosphate ester prodrug [83,84], and is rapidly generated during the absorption process, and in blood following oral or intravenous administration of TR-701 [83]. Preliminary reports have shown that torezolid was 4-fold more active than linezolid against staphylococci and enterococci. The drug is in Phase 3 clinical trials for treatment of hospital and community-acquired infections including for the treatment of severe complicated skin and skin structure infections caused by gram-positive bacteria, especially drug-resistant strains such as methicillin-resistant *Staphylococcus aureus* (MRSA), and community-associated pneumonia [44,85].

An investigational pyrrolopyrazolyl-substituted oxazolidinone, RWJ-416457, discovered at Johnson & Johnson (Raritan, NJ, USA) inhibited the growth of linezolid-susceptible staphylococci, enterococci and streptococci at concentrations of ≤4 mg/L, generally exhibiting twofold to fourfold greater potency than that of linezolid [86]. Recent data on a murine infection model demonstrated that the drugs has an *in vivo* activity consistent with its *in vitro* potency, relative to *S. aureus* and *S. pneumoniae* infections. All together these data support further clinical evaluation for the treatment of SSTIs [87]. 

Using a combination of structural information and computational analysis, Rib-X Pharmaceutical developed a new oxazolidinone family, Rχ-01. Recent data on this family of oxazolidinones show that it has a greater affinity (at concentrations more than 100 times lower than that for linezolid) to the ribosome and therefore a greater intrinsic activity against linezolid-resistant isolates and can combat major causative agents in the community such as streptococci, *Moraxella* or *Haemophilus*. A member of the Rχ-01 family of compounds is currently undergoing clinical trials [88], and its derivatives RBX-8700 and ranbezolid (RBX-7644), developed by Ranbaxy Research Laboratory (New Delhi, India), were identified as agents against MDR-*M. tubercolosis* [89]. RBX-8700, in particular, possesses a potent antibacterial and concentration dependent activity against all slowing mycobacteria [90]. Ranbezolid has shown an excellent anti-anaerobe activity [91].

## 10. Conclusion

Linezolid is still a very active antibiotic and its value to address serious emerging resistance among Gram-positive cocci has been well documented. Antimicrobial resistance problems in which linezolid appears therapeutically suited continue to be severe infections sustained by MDR MRSA and enterococci. These positive features must be balanced against the safety profile and the possibility of emerging linezolid resistance. This threat has become real especially under prolonged therapies. 

Furthermore the recent acquisition of a linezolid resistance mechanism based on a modification of A2503 and mediated by the *cfr* gene localized on transferable elements, indicates a potential to disseminate among Gram-positive pathogenic strains. This gene, originally found in animal strains, is now present clinically. Attention should be paid to the fact that these strains might also be selected under treatment with phenicols or macrolides, and this could be due to co-selection, might multiply the risk of development of linezolid-resistant strains.
